# Safety and efficacy of the combination of T-DM1 with radiotherapy of the central nervous system in a patient with HER2-positive metastatic breast cancer: case study and review of the literature

**DOI:** 10.3332/ecancer.2015.586

**Published:** 2015-10-22

**Authors:** Giuliano Santos Borges, Rodrigo Kraft Rovere, Stéphanie Mereniuk Kappel Dias, Fernando Henrique Chong, Mayara dos Santos Morais

**Affiliations:** 1 Médico Novos Tratamentos Litoral (Clínica de Neoplasias Litoral), Itajaí, Santa Catarina, CRM-SC 11867, Brazil; 2 Médico Hospital Santo Antônio, Blumenau, Santa Catarina, CRM-SC 15751, Brazil; 3 Médica Prefeitura Municipal de Balneário Camboriú, Santa Catarina, CRM-SC 21977, Brazil; 4 Médico Plantonista, CRM-SC 21967, Brazil; 5 Médica Prefeitura Municipal de Apiúna, Santa Catarina, CRM-SC 21955, Brazil

**Keywords:** breast cancer, HER2-positive, TDM1, radiotherapy

## Abstract

Approximately 35% of patients with confirmed HER2 breast cancer progress to metastases of the central nervous system (CNS). Total cerebral radiotherapy is considered as standard treatment for these cases; however, studies have shown that some chemotherapy drugs can be used during radiotherapy without significantly increasing its toxicity. In this article, we report the case of a patient with HER2-positive breast cancer who showed isolated progression of the illness in the CNS, which was observed during the treatment period using T-DM1 concomitantly with radiotherapy of the CNS without apparent toxicity of the combination and keeping the illness controlled. Through a review of the literature on the use of radiotherapy and chemotherapy with T-DM1 for the treatment of cerebral metastases in HER2-positive breast cancer, we describe the efficacy and tolerance of the concomitant application of these treatments.

## Introduction

Breast cancer is one of the most common neoplasia in Western countries and one of the main causes of death among women, as well as the most important among women from 40 to 59 years of age [1]. Of the total cases of mammary neoplasia, 15 to 20% are HER2 positive [2].

Generally, among patients with breast cancer, approximately 25% progress to metastases of the central nervous system (CNS) [3]. Meanwhile, among those with confirmed HER2, this number increases to 37.3% [4]. The management of metastatic cerebral lesions is multimodal and depends on a series of factors, such as age, general health of the patient, survival time, demonstrated symptomology, intensity of cerebral involvement (number, size, and location of the lesions), state of the systemic illness, as well as the preference of the patient. While surgery and radiosurgery may be treatment options for a small number of lesions, total cerebral radiotherapy continues to be the standard when the illness is multifocal. Independently of the management strategy, the main goal of treatment is to preserve the neurological status of the patient at its highest level, thus improving their general well-being and increasing their survival [3, 5].

It is known that the blood–brain barrier limits the penetration of the anti-neoplasic agents into the CNS. Nevertheless, the true functioning of this system in metastatic illness is not completely understood, given the capacity of these lesions to take up contrast in imaging studies, suggesting a breach in this barrier [6]. Consequently, the use of systemic agents has demonstrated intracranial responses in chemosensitive tumours, and thus, the ability to increase the effects of radiotherapy in more resistant tumours [3].

In this study, we describe the efficacy and tolerance of the treatment of a patient with HER2-positive breast cancer with metastases of the CNS subjected to total cerebral radiotherapy simultaneously with systemic therapy with trastuzumab emtansine (T-DM1).

## Case study

A 62-year-old female patient with leucoderma, with a diagnosis of carcinoma of the breast, was subjected to modified radical left mastectomy and axillary lymphadenectomy, followed by breast reconstruction with a prosthesis.

An anatamopathological study showed a 2 cm invasive ductal carcinoma in the left breast, the presence of micrometastases in a lymph node, positivity for oestrogen and progesterone receptors and a score of 3 + for the product of the HER2 oncogene. The pathological staging observed was pT1cpN1miM0.

Chemotherapy was performed with four cycles of 60 mg/m^2^ of adriamycin and 600 mg/m^2^ of cyclophosphamide within a period of 2 months. Afterward, trastuzumab and paclitaxel were initiated, but the patient completed only two cycles, as this was interrupted by an allergic reaction to taxane. The use of trastuzumab and tamoxifen was continued for one year.

Due to bone pain 7 months after the start of treatment, a bone scan was requested, which revealed increased osteoblastic activity in the spinal column at T6 and T7.

Computed tomograpghy (CT) scans of the thorax, abdomen, pelvis, and spine showed pulmonary micronodules and a lytic lesion with a component of soft areas affecting the pedicle and elements of the posterior arch of T6, compatible with neoplastic implant.

The patient developed symptoms compatible with medullary compression. Magnetic resonance imaging (MRIs) of the cervical and thoracic spine showed secondary implants in T6 and at the level of C3 and C5, with components of soft areas projecting into the epidural space, reducing the width of the spinal canal and discreetly compromising the medulla in this region.

The anatamopathological study of the bone lesion confirmed metastatic adenocarcinoma in the bone tissue. The immunohistochemical analysis indicated the breast as the probably site of origin, with positive oestrogen receptors and a score of 3 + for the product of the HER2 oncogene.

Thus, treatment with radiotherapy and fulvestrant associated with zoledronic acid was initiated and maintained for 4 months.

With the clinical progression, evidenced by an increase in the pain intensity, weekly docetaxel was initiated, followed by capecitabine and lapatinib for a period of 1 month; however, the symptoms did not improve.

She was referred to protocol NCT01419197, a randomised, open (TH3RESA), Phase-III, multicentric study with two branches, which assessed the efficacy and safety of T-DM1 in comparison with other treatments in patients with HER2-positive metastatic or locally advanced/recurrent breast cancer. The patients in question were randomised into the group which would be treated with T-DM1.

Then, chemotherapy cycles with T-DM1 every 21 days were started. In the following visits and throughout the cycles, the bone pain was unchanged with the use of morphine.

During the treatment with T-DM1, imaging studies were done every 6 weeks for control. From the 12th week of treatment with the protocol, CT scans of the thorax, abdomen, and spine showed a partial response, with the disappearance of the pulmonary implants, and only the bone lesions remaining. There was no evidence of progression of the illness.

In addition, Doppler echocardiograms were taken every 3 months which showed no cardiac toxicity on the treatment with T-DM1.

In the first cycle, mild nausea was reported, possibly related to the treatment with T-DM1, which was treated with 10 mg bromopride orally 3× per day.

In the fourth cycle, there were signs of moderate neuropathy, possibly related to T-DM1, and 75 mg Lyrica orally per day was started to treat this condition.

Eleven months after the start of treatment with T-DM1, she had a convulsive episode. A cranial MRI was performed, which showed isolated progression in the CNS. The results were discussed with the study team and it was decided to continue the treatment with T-DM1, as the patient showed controlled illness outside the CNS. Thus, total cerebral radiotherapy was initiated, applied to the CNS with a dose of 3 Gy/dau, concomitant with the treatment with T-DM1. The use of 10 mg hidantal orally 3× per day was instituted for the control of convulsive symptoms.

Following 6 months of monitoring, it was observed that the patient had a good tolerance for radiotherapy, as no adverse reactions or clinically significant toxicity were observed. A control MRI was performed, which showed a lack of progression in the CNS. Other imaging studies continued to show a partial response outside the CNS. No sequelae appeared following the treatment.

This case suggests that in a patient with isolated progression of metastatic illness in the CNS, a treatment option would be the administration of radiotherapy concomitantly with treatment with T-DM1, as no significant toxicity was shown and there was control of the progression of the illness.

## Discussion

Cerebral metastases are the most common form of intracranial neoplasia, as they are present in up to 25% of all patients with advanced cancer. In breast cancer, the incidence is approximately 25%, and it is the second most common type to evolve with metastases in the CNS, after only pulmonary neoplasias. HER2 positivity is recognised as a risk factor for cerebral metastases in patients with breast cancer, increasing the incidence of these metastases to 37.3% [2, 4, 7, 8].

Due to the accuracy and sensitivity of current diagnostic methods, an increase in the number of cases of metastases from breast cancer has been observed. The principal sites for breast cancer metastases are the CNS, with the parenchyma being the most affected area, followed by the cerebellum and the encephalic trunk. The supratentorial region is more affected than the infratentorial, with a predilection for perivascular areas and connections between white and grey matter. Metastatic lesions located in the infratentorial region generally originate from malignancies located in the abdominal or pelvic regions, once they gain access through the venous drainage of these areas through the internal vertebral venous plexus–Batson plexus [7].

HER-2 positivity confers an increased risk of recurrence of distant metastases and a decrease in the survival of patients with breast cancer without anti-HER2 treatment, while there is an increase in survival with the institution of this treatment. Another point is the association with the aggressiveness of metastases, which is elevated in HER2-positive patients. In most cases, dissemination follows the identification of other systemic lesions. Trastuzumab, an anti-HER2 antibody, has a limited ability to cross the blood–brain barrier due to its high molecular weight. Due to this difficulty, approximately 10% of patients taking trastuzumab exclusively as a treatment for metastatic breast cancer develop isolated involvement of the CNS [6].

Multiple cerebral metastases of breast cancer exist in two-thirds of patients at the time of diagnosis, and they generally have a random distribution, and can produce any neurological sign or symptom, or a combination of these. The principal clinical manifestations are headaches, behavioural changes, cognitive changes, and clinical signs such as aphasia, difficulty walking, focal deficits and epileptic crises. Nevertheless, 20% of cerebral metastases remain asymptomatic [7].

The most effective method for complementary assessment is magnetic resonance imaging (MRI) with gadolinium contrast. This has greater sensitivity than computerised tomography (CT) with contrast for the identification of lesions in the parenchyma or leptomeninges. Approximately 20% of patients with a single lesion detected by CT are found to have multiple lesions when assessed by MRI. [8]

Treatment options for patients with cerebral metastases of breast cancer are the use of systemic chemotherapy, total or localised cerebral radiotherapy, conventional surgery, and radiosurgery [5]. Systemic chemotherapy is only an option for some metastatic breast cancers. In cases with multiple cerebral metastases, which correspond to most cases of breast cancer, the best option is total or localised cerebral radiotherapy. Conventional surgery is indicated in patients with a single, surgically accessible metastasis, while for surgically inaccessible or small lesions, there is a more recent means of treatment, which is stereotactic radiosurgery, which is also an option for salvage treatment following prior treatment with surgery of total cerebral radiotherapy, or as an adjuvant treatment following resection of a single metastasis [5, 9]. The adverse effects associated with radiotherapy applied to the CNS are varied, such as headache, convulsions, alopecia, fatigue, skin rashes, nausea, and vomiting during the initial treatment period. Later, ataxia, urinary incontinence, and cognitive or memory-related disturbances may be observed. An average gain of 3 to 6 months in patient survival is achieved, along with a good response relative to the quality of life, as well as a radiological response of nearly 60%. There is still no consensus on the best dose of this therapy, as more studies are needed to find the optimum dose weighing the risks and benefits. When there is some difficulty, studies have shown that use of a dose less than 3 Gy/fraction achieves a good response in patients with breast cancer that is sensitive to radiotherapy and those with a life expectancy greater than 6 months [10, 11].

The use of chemotherapy in the treatment of advanced breast cancer depends on multiple factors. In addition to a good doctor–patient relationship, in which both must be in agreement on the best course of treatment within their expectations and risk factors, the characteristics of the patient and his or her type of cancer are important to the decision on what drug should be used. The greatest difficulty in cancer management is in establishing the best time for the use of medication. In this case, trastuzumab–emtansine was used, better known by the term T-DM1. The drug is made up of a combination of three parts: a specific humanised antibody for the extracellular region of the HER2 receptor – trastuzumab – an antimicrotubule agent derived from maytansine – DM1 – and a linking thioether responsible for connecting the two structures already mentioned. As for its mechanism, trastuzumab links to the HER2 receptor, allowing for the internalisation of the T-DM1 molecule by the endosomes, mediated by antigens. Then, the DM1 portion is released in the intracellular environment. Then, this reacts by inhibiting the polymerisation of beta-tubuline. This occurs through the linking process, which occurs in a competitive manner with the linking alkaloid. Following this process, cellular death occurs [12].

As it is a relatively new drug, its interactions with other medications are still being investigated. In a study of the association of T-DM1 with pertuzumab, the risk of complications was shown to be low. As for the association of T-DM1 with taxanes (taclitaxel and docetaxel), studies are being carried out to find possible adverse reactions of clinical importance. Until then, it is demonstrated that there is no relevant difference between the isolated administration of T-DM1 and its association with taxanes in relation to the severity and types of adverse reactions. As a monotherapy, T-DM1 showed an increase in survival and lower toxicity in relation to the use of lapatinib plus capecitabine in patients with advanced HER2-positive breast cancer in prior treatment with trastuzumab and taxanes [13, 14].

While it has shown good clinical results, T-DM1 is not a drug which is free of side effects. Among the main side effects were fatigue (64.5), nausea (48.4%), headache (30.4%), fever (28.4%), nosebleeds (28.9%), and constipation (28.6). These events were classified with grade 1 or 2 in intensity. It is also important to indicate the events classified with grade 3 or higher, of which the main effects were thrombocytopenia (7.7%), hypocalcaemia (4%), increased fatigue (4%), increased AST (3.3%), shortness of breath (2.9%), anaemia (2.6%), cellulite (2.6%), hyperglycaemia (2.2%), and low back pain (2.2%) [15].

The published studies still show controversies relating to the efficacy of treatment with isolated trastuzumab in advanced breast cancer with cerebral metastasis. What has been discussed is that the increase in the survival rate may be related to control of the systemic illness caused by the drug and not by the control of the cerebral metastasis [16].

In the case study done by Torres *et al*, a regression of the size of multiple asymptomatic metastases of the CNS was observed, affecting cerebral and cerebellar hemispheres and the cerebral trunk in a young patient with advanced inflammatory mammary neoplasia following treatment with Ado-trastuzumab emtansine [17]. Similarly, in the report of Torres *et al*, an unexpected reduction in the size of metastases in the right parietal lobe and adjacent leptomeninges and cerebellum were observed bilaterally following additional total cerebral radiotherapy and treatment with T-DM1 for the control of the systemic illness [18].

## Conclusion

We conclude from this study that there was a good response to the association of total cerebral radiotherapy to the use of T-DM1, keeping the patient free from the progression of the illness in the CNS, with good tolerance and with no evidence of adverse reactions, clinically significant toxicity, or sequelae.

## Conflicts of interest

We declare no conflicts of interest of any type in the preparation of this manuscript.
